# Debate on industry influence on OH organizations/T.K. Joshi's tantrums!

**DOI:** 10.4103/0019-5278.40819

**Published:** 2008-04

**Authors:** Ramnik Parekh

**Affiliations:** Former President, IAOH. E-mail: occhealthindia@yahoogroups.co.uk

Sir,

T.K. Joshi's letter in *International Journal of Occupational and Environmental Health* (*IJOEH*) is amusing and equally annoying to some of us who are closely connected with Indian Association of Occupational Health (IAOH) for several decades. He has been invited to a number of important meetings and has been even honoured by the same organization as late as this year when he was awarded the most coveted ADM oration! As a matter of fact, the IAOH has always gone out of the way and often at the displeasure of the asbestos industry to invite Dr. Joshi to candidly air his opinions, which he has done in our conferences, particularly in one seminar when I was the Chair for a debate on asbestosis. While he claims in his letter that no one from IAOH came to his rescue and thanks two gentlemen specifically for that kind favour; he sent several e-mail messages to many of us thanking us profusely for our support to his cause and for helping in protecting his job!

Therefore now, without even ever discussing the subject with the office-bearers of IAOH till date, he has chosen to pose himself as “a martyr” for the cause in the international community or to appease Ban Asbestos lobby for the reasons only known to him. I am also surprised that a reputed journal like *IJOEH* is letting disgruntled professionals use it as a forum to achieve their personal agendas. I wish he had gathered enough courage to discuss with IAOH office-bearers about this subject and the issues he has raised vehemently in an international journal. I can only sympathize with him at this moment for the lack of courage.

